# Isolation and in vitro antimicrobial susceptibility of porcine *Lawsonia intracellularis* from Brazil and Thailand

**DOI:** 10.1186/s12866-019-1397-7

**Published:** 2019-01-31

**Authors:** Suphot Wattanaphansak, Carlos Eduardo Real Pereira, Wenika Kaenson, Pornchalit Assavacheep, Rachod Tantilertcharoen, Talita Pilar Resende, Javier Alberto Barrera-Zarate, Juliana Saes Vilaça de Oliveira-Lee, Ulrich Klein, Connie Jane Gebhart, Roberto Maurício Carvalho Guedes

**Affiliations:** 1 Department of Clinic and Surgery, Veterinary School, Universidade Federal de Minas Gerais, PO Box 567, Belo Horizonte, Minas Gerais 31270-901 Brazil; 2 Department of Veterinary and Biomedical Sciences, College of Veterinary Medicine, University of Minnesota, St. Paul, MN 55108 USA; 3 Department of Veterinary Medicine, Faculty of Veterinary Science, Chulalongkorn University, Pathumwan, Bangkok, 10330 Thailand; 4 Elanco Animal Health Inc., Mattenstrasse 24A, 4058 Basel, Switzerland

**Keywords:** Antimicrobial susceptibility, Ileitis, *Lawsonia intracellularis*, MIC, Proliferative enteropathy, Pigs

## Abstract

**Background:**

*Lawsonia intracellularis* is an obligate intracellular bacterium which cannot be cultured by conventional bacteriological methods. Furthermore, *L. intracellularis* needs enriched medium and a unique atmosphere for isolation, cultivation and propagation. Because of this,there are only a few isolates of *L. intracellularis* available and few studies in vitro demonstrating the susceptibility of this bacterium to antimicrobial agents. The objectives of this study were to isolate South American and Southeast Asia strains of *L.intracellularis* and to determine the in vitro antimicrobial activity against these isolates. Tested antimicrobials included: chlortetracycline, lincomycin, tiamulin, tylosin and valnemulin(against both Brazilian and Thailand strains) and additionally, amoxicillin, zinc-bacitracin, carbadox, enrofloxacin, gentamicin, sulfamethazine, trimethoprim, spectinomycin and a combination (1:1) of spectinomycin and lincomycin were also tested against the Thai isolates. The minimum inhibitory concentration (MIC) was determined by the antimicrobial activity that inhibited 99% of *L. intracellularis* growth in a cell culture as compared to the control (antimicrobial-free).

**Results:**

Two strains from Brazil and three strains from Thailand were successfully isolated and established in cell culture. Each antimicrobial was evaluated for intracellular and extracellular activity. Pleuromutilin group (valnemulin and tiamulin) and carbadox were the most active against *L. intracellularis* strains tested. Tylosin showed intermediate activity, chlortetracycline had variable results between low and intermediate activity, as well as spectinomycin, spectinomycin and lincomycin, amoxicillin, sulfamethazine and enrofloxacin. *L. intracellularis* was resistant to lincomycin, gentamicin, trimethoprim, colistin and bacitracin in in vitro conditions.

**Conclusions:**

This is the first report of isolation of *L. intracellularis* strains from South America and Southeast Asia and characterization of the antimicrobial susceptibility patterns of these new strains.

## Background

Proliferative enteropathy (PE), or ileitis, is one of the most important enteric bacterial infectious diseases in grower and finisher pigs. PE was first recognized in the North American swine industry in the early 1930s [1]. Since then, PE has become a major enteric health concern for swine production in the United States and around the world [1]. The etiological agent of this disease is an obligate intracellular, microaerophilic and Gram-negative bacterium named *Lawsonia intracellularis* [1]. There are two different clinical syndromes commonly seen with PE, acute and chronic. The acute form is characterized by hemorrhagic diarrhea and occasional sudden death and occurs in adult pigs. The chronic form is observed in young pigs which commonly exhibit diarrhea, anorexia, and poor growth [2]. There are two main forms of PE control, vaccination and antimicrobial agents. Vaccination has demonstrated good efficacy and, alternatively, antimicrobial therapy is a more immediate effective strategy [3]. In the swine industry, prophylactic antimicrobial therapy can be used through feed or water.

When a PE outbreak occurs in a herd, antimicrobial therapy is often used to control the disease [3]. Antimicrobial therapy with an effective antimicrobial agent is able to stop the progression of the PE outbreak in a short period of time [3]. Therefore, antimicrobial selection is critical for achieving the best possible outcome for the herd. Despite the importance of the antimicrobial treatment for PE, little information about in vitro sensitivity results against *L. intracellularis* for antimicrobial selection is available [4–6]. The main reason for this lack of information is due to the difficulty in isolating *L. intracellularis* from infected intestine or fecal samples, requiring experienced personnel and several months for the establishment of a pure *L. intracellularis* culture. Consequently, the in vitro sensitivities of *L. intracellularis *for antimicrobials are difficult to obtain in a timely fashion to treat a PE outbreak. Furthermore, the obligate intracellular nature of *L. intracellularis *prohibits the use of standard antimicrobial susceptibility testing methods. Instead, a complicated tissue culture system has been used to evaluate antimicrobial activity against some isolates of *L. intracellularis* originated from the United States and two other countries [4–7].

A previous study found that isolates of *L. intracellularis* can have different antimicrobial sensitivities [6]. Therefore, selection of antimicrobials for which most isolates showed good response would yield a better treatment success. So far, there is no information about thein vitro sensitivities of *L. intracellularis* isolated from Latin America and Southeast Asia, where swine production is an important industry and there is documented high prevalence of proliferative enteropathy in these areas [5, 8–11]. In order to expand the limited information on in vitro antimicrobial sensitivity against *L. intracellularis*, additional primary isolates of *L. intracellularis* from Brazil and Thailand must be obtained, propagated *in vitro* and then evaluated.

The overall aims of this investigation were to obtain new isolates of *L. intracellularis* from Latin America and Southeast Asia and to determine the *in vitro* minimum inhibitory concentration (MIC) of antimicrobials against these new isolates for use as a guideline for antimicrobial selection in the treatment and control of PE.

## Results

*L. intracellularis* strains BRPHE01_E5, BRPHE02_E8,CUPHE01_SW13, CUPIA01_SW13, and CUPIA02_SW13 were successfully isolated as pure cultures from swine intestines affected by PE. The number of cells heavily infected by each isolate, which is an indicator of viable bacteria, dramatically increased to approximately 100% around passage 5. Moreover, all isolates were continuously maintained and propagated *in vitro.*

The *L.intracellularis* isolates were tested for antimicrobial MICs at passages up to 15. The final concentration of inoculum was approximately between 10^6^ and 10^7^*L. intracellularis/*ml for all isolates.

### Brazilian isolates

**Fig. 1:**
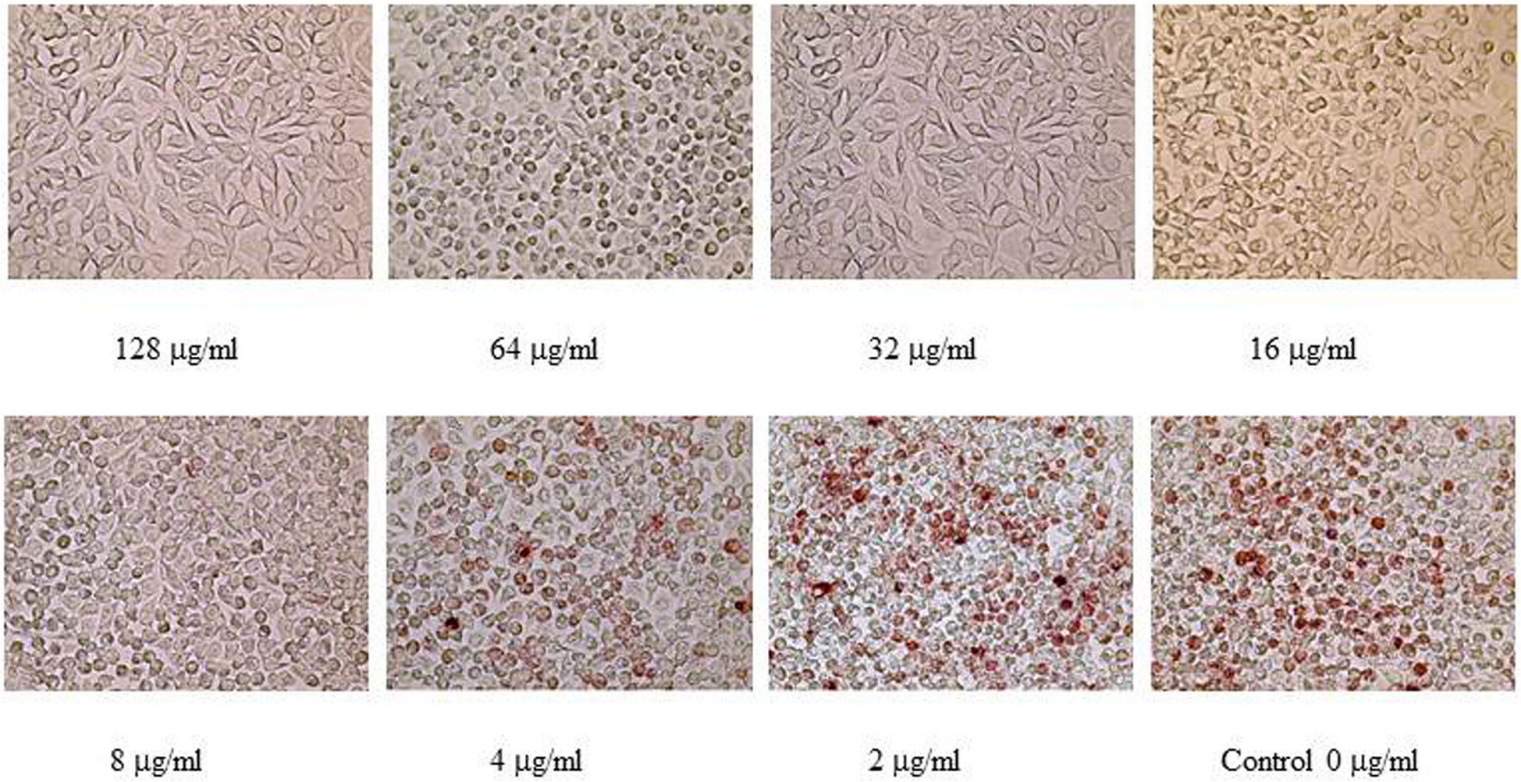
An example of an MIC endpoint for tiamulin against *L. intracellularis* strain CUHE01_SW13 at passage 6. Photographs of McCoy cells infected with *L. intracellularis* growing in the presence of tiamulin with concentrations ranging from 2 μg/ml to 128 μg/ml. There was no *L. intracellularis* growth in cells treated with tiamulin at concentrations ranging from 16 μg/ml to 128 μg/ml. The numbers of HICs dramatically increased at the concentration of 4 μg/ml (> 1% compared to control). Therefore, the MIC of tiamulin for this *L. intracellularis* strain is 8 μg/ml (< 1% compared to control)

The value of extracellular and intracellular MICs of all tested antimicrobials against the Brazilian *L. intracellularis* isolates are shown in Table 1. Compared to the antimicrobial-free control, the MIC endpoints for each antimicrobial were the concentrations that were able to inhibit 99% of *L. intracellularis* proliferation. An example of heavily infected cells (HIC) of *L. intracellularis*growth at different concentrations of antimicrobials in the McCoy cells is shown in Fig. 1. The difference in MIC median of two independent preparations for each isolate was within two-fold dilutions.

**Table 1:** Extracellular and intracellular MIC endpoints for 5 antimicrobials against two Brazilian *L. intracellularis* isolates. The bacteria were prepared independently and tested twice. The endpoint was obtained from 3 replicates of each passage. Minimum Inhibitory Concentration (MIC) μg/ml

Antimicrobials	Minimum Inhibitory Concentration (MIC) μg/ml			
	BRPHE01_E5 Passages 13–14		BRPHE02_E8 Passages13–14	
	Intracellular activity	Extracellular activity	Intracellular activity	Extracellular activity
1. Chlortetracycline	32–64	32–64	8–16	64
2. Lincomycin	> 128	> 128	> 128	> 128
3. Tiamulin	≤0.125	1–2	1–2	0.5
4. Tylosin	2–8	> 128	2	16–32
5. Valnemulin	≤0.125	≤0.125	≤0.125	≤0.125

For intracellular MIC testing, tiamulin and valnemulin had the highest activity against the Brazilian *L. intracellularis* isolates, with MICs rangingfrom≤0.125 to 2 μg/ml. Tylosin showed moderate activity against the *L. intracellularis* isolates with MICs rangingfrom 2 to 8 μg/ml. Chlortetracycline had lower activity with MICs rangingfrom 8 to 64 μg/ml. Lincomycin showed the lowest activity against the *L. intracellularis* isolates with MICsgreater than 128 μg/ml.

For extracellular activity, the results showed that tiamulin and valnemulin had highest activity against the *L. intracellularis *isolates with MICs from ≤0.125 to 2 μg/ml. Chlortetracycline had moderate antimicrobial activities against *L. intracellularis* with MICs ranging from of 32 to64 μg/ml. Tylosin had different results between the two strains, with moderate activity against BRPHE02_E8 (16–32 μg/ml) and low activity against BRPHE01_E5 (> 128 μg/ml). Lincomycin had lowest antimicrobial activities with the MIC of > 128 μg/ml for both isolates.

### Thailand isolates

The median value of extracellular and intracellular MICs for all tested antimicrobials against three Thailand *L. intracellularis* isolates is shown in Table 2. For intracellular MIC testing, carbadox, tiamulin and valnemulin displayed the highest activity against all three Thailand *L. intracellularis* isolates with MICs of ≤0.125 to 1 μg/ml. Amoxicillin, enrofloxacin and tylosin showed moderate activity against all three *L. intracellularis* isolates with MICs rangingfrom 2 to 32 μg/ml. Zinc-bacitracin, chlortetracycline, colistin, gentamicin, lincomycin, spectinomycin, lincomycin:spectinomycin (1:1), sulfamethazine and trimethoprim showed the lowest activity against all three *L. intracellularis* isolates with MIC ranging from 64 to > 128 μg/ml.

**Table 2:** Extracellular and intracellular MIC endpoints for 15 antimicrobials against 3 Thailand *L. intracellularis* isolates. The bacteria were prepared independently and tested twice. The endpoint was obtained from the median value of 3 replicates of each passage

Antimicrobials	Minimum Inhibitory Concentration (MIC) μg/ml					
	CUPHE01_SW13 Passages 6–7		CUPIA01_SW13 Passages 8–9		CUPIA02_SW13 Passages 5–6	
	Intracellular activity	Extracellular activity	Intracellular activity	Extracellular activity	Intracellular activity	Extracellular activity
1. Amoxicillin	8–16	> 128	8	> 128	16–32	64
2. Carbadox	≤0.125	8	≤0.125–0.25	0.5–1	≤0.125–0.25	0.5
3. Chlortetracycline	> 128–64	> 128	> 128	> 128	32	32
4. Colistin	> 128	> 128	> 128	> 128	> 128	> 128
5. Enrofloxacin	8	> 128	4–8	64	16	32–64
6. Lincomycin	> 128	> 128	> 128	> 128	> 128	> 128
7.Lincomycin+Spectinomycin	128–64	32	> 128	8–4	64–128	2
8. Gentamicin	> 128	> 128	> 128	128- > 128	> 128	64–128
9. Spectinomycin	128	32	> 128	8–4	> 128–128	4–2
10. Sulfamethazine	128	> 128	4–8	> 128	32–64	> 128
11. Tiamulin	1	8	1	4	≤0.125–0.25	0.5
12. Trimethoprim	64	> 128	> 128–128	> 128	> 128	> 128
13. Tylosin	8–16	32	4	64	2	8
14. Valnemulin	≤0.125	0.5–1	≤0.125	0.5–0.25	≤0.125	0.25
15. Zinc-Bacitracin	> 128–128	> 128	> 128	> 128	> 128	> 128

For extracellular activity, the results showed that carbadox, tiamulin, and valnemulin had highest activity against the three *L. intracellularis* isolates with MICs from 0.25 to 8 μg/ml. Spectinomycin, lincomycin:spectinomycin (1:1), and tylosin had moderate antimicrobial activities against *L. intracellularis* with MICs of from 2 to 32 μg/ml. Amoxicillin, bacitracin, chlortetracycline, colistin, enrofloxacin, gentamicin, sulfamethazine and trimethoprim had lowest antimicrobial activities, with the MICs ranging from 64 to 128 μg/ml.

## Discussion

Although outbreaks of PE occur globally, it has been estimated that less than 25 *L. intracellularis* isolates have been successfully cultured and maintained *in vitro* worldwide. Of these, only 15 *L. intracellularis* isolates have been tested for their antimicrobial susceptibilities:three United Kingdom isolates [4, 7]; one Danish, six North American isolates [5]; and two Korean isolates [6]. Our study was the first to culture *L. intracellularis* from PE field cases and to evaluate its antimicrobial susceptibility in Brazil and Thailand. Five *L. intracellularis* isolates were successfully established in pure culture, two from Brazil and three from Thailand. Like other continents, a high level of *L. intracellularis* infection had been found in swine herds in South American and Southeast Asian countries. It was estimated that the herd prevalence of PE in pigs was 77% in Vietnam, 85% in China, 86% in the Philippines, 94% in Japan, and 100% in Korea, Malaysian and Thailand [8]. Serological studies conducted in Brazil and Thailand found that, in absence of herd vaccination, 100% of commercial herds were seropositive for *L. intracellularis* infectionindicating direct contact with the pathogen [10, 12].

Studies describing *L. intracellularis* susceptibility to antimicrobials are limited. Published data have shown MIC endpoints for diverse isolates expressed as both extracellular and intracellular MICs using a tissue culture system [4–7]. Both MIC endpoints were designed to mimic the pattern of *L. intracellularis* infection in vivo. The bacteria would be exposed to antimicrobials before and after invasion into intestinal cells (extracellularly and intracellularly, respectively). Similar to previous studies [4–7], our MIC endpoints for the two independent bacterial preparations (intracellular and extracellular) consistently fell within two-fold dilution, indicating the reproducibility of the assays.

Our extracellular and intracellular MIC results showed that carbadox, tiamulin and valnemulin were the most active compounds against the Brazilian and Thai isolates, inhibiting extracellular and intracellular activities with concentrations of ≤0.125–8 μg/ml. Since the use of carbadox is not permitted in Brazil, this component was not tested for the Brazilian isolates. Tylosin had intermediate activity against all the isolates with MICs ranging from 2 to 64 μg/ml for intracellular and extracellular activities, except for one Brazilian isolate, which had an MIC of > 128 μg/ml for extracellular activity. Lincomycin was the least active compound against the Brazilian and Thai *L. intracellularis* isolates with an MIC of > 128 μg/ml. This might be due to the fact that this antimicrobial has been used more intensively with high dosages for control of other endemic pathogens in swine farms or by the difficulty of extrapolating the in vitro results to the in vivo scenario.

When compared to other data, the MIC values for the Brazilian and Thai *L. intracellularis* isolates tended to have higher MIC endpoints than the North American, European and Korean isolates [5, 6]. For intracellular MIC results, valnemulin and tiamulin demonstrated the highest activity against the Brazilian and Thai *L. intracellularis* isolates, similar to the results previously published for North American, European, and Korean isolates [5, 6].

Chlortetracycline showed intermediate activity against Brazilian isolates (8–64 μg/ml), whereas Thai isolates less sensitive (64 - > 128 μg/ml). This was the only antibiotic with substantial differences between the Thai and Brazilian isolates, with the exception of the Thai strain CUPIA02_SW13, which had similar results to the Brazilian isolates and to previous studies [4–6] that chlortetracycline ranged from 0.125 μg/ml in one USA strain [5] to 64 μg/ml in Korean and European isolates [4, 6]. Thai isolates were also resistant to colistin, gentamicin, trimethoprim and bacitracin (64 - > 128 μg/ml).

Previous reports have shown that extracellular MICs for all tested antimicrobials were higher than the intracellular MICs [5, 6], and our results are similar. The difference between intracellular and extracellular MICs may be due to the period of time *L. intracellularis* was exposed to the antimicrobial agent in each of the preparations, the MIC assays were performed as described by Wattanaphansak et al. [5]. Extracellular MICs were designed to have a 24 h incubation, while intracellular preparations were incubated with *L.intracellularis* for three consecutive days. Moreover, it is likely that the effect of antimicrobials accumulated inside the cells overtime. This accumulation of intracellular antimicrobial concentration suggests that a one-time antimicrobial treatment may be insufficient to inhibit the growth of *L. intracellularis*in vitro.

## Conclusion

In conclusion, our in vitro data expand the antimicrobial susceptibility information for *L. intracellularis* generated for isolates from pig farms worldwide. Based on our *in vitro* results, we confirm that Brazilian and Thai *L. intracellularis* isolates have a unique *in vitro *antimicrobial sensitivity pattern, in relation to other regions. Since it is impractical to culture *L.intracellularis* and perform an antimicrobial sensitivity test during a PE outbreak, our data serve as a guideline for the range of antimicrobial activities against *L. intracellularis*.

## Methods

### *L. intracellularis* isolation

#### Brazilian isolates

*L. intracellularis* isolates were obtained from pigs affected with the acute form of PE. For the first isolate,BRPHE01_E5, ileum was obtained from adiarrheic finishing pig from a multi-site commercial farm in the metropolitan region of Belo Horizonte, Minas Gerais state, Brazil, in 2011. The second isolate, BRPHE02_E8, was obtained from a diarrheic finishing pig from a herd located in São Paulo state, Brazil, also in 2011. Affected intestines were submitted to the Veterinary Pathology Laboratory at the Universidade Federal de Minas Gerais, for routine bacteriology examination and immunohistochemistry confirmation of *L. intracellularis*, which was the only etiologic agent found.

#### Thailand isolates

Three PE affected swine intestines were used to obtain *L. intracellularis* isolates. One intestine had the acute form of the disease, characterized by blood clots in the lumen associated with thickening of the small intestine mucosa, proliferative hemorrhagic enteropathy (PHE), and two hadthe chronic form of the disease, characterized by the thickening of the small intestine mucosa, porcine intestinal adenomatosis (PIA). The intestines were collected from three distinct herds in the Western region of Thailand. The PHE strain, CUPHE01_SW13, was obtained from a gilt that died suddenly with acute bloody diarrhea in a breeding herd in Kanchanaburi province in 2013. Both of the PIA intestines, CUPIA01_SW13 and CUPIA02_SW13, were collected from finishing pigs at the slaughter house in Nakornpathom province in 2012. All three affected intestines were submitted to the Veterinary Diagnostic Laboratory at the Chulalongkorn University, Nakhonpathom, for PCR confirmation of *L. intracellularis* infection. All three infected intestine samples were PCR positive for *L. intracellularis *which was the only etiologic agent found.

#### Isolation protocol

Infected segments of the jejunum or ileum were cut into several pieces of approximately 5 cm and kept at -80 °C until the initiation of the bacterial isolation process. The primary isolation of *L. intracellularis* from the infected intestines was modified from a previous study [11]. Briefly, the mucosa from 5 cm of infected intestines was scraped and blended by using a tissue grinder. The blended mucosa was suspended in 40 ml of sterile phosphate buffered saline (PBS). The suspension was centrifuged at 500 g for 20 min and the supernatant was filtered sequentially through 70 μm, 5 μm, and 0.8 μm filters. The filtered suspension was then centrifuged at 5000 g for 20 min. The pellet was re-suspended in fresh culture medium containing: 50 μg/ml gentamicin and 10 μg/ml vancomycin. The bacterial suspension was transferred to 1-day-old McCoy cells and incubated in sealed bags with 10:10:80 CO_2_:H_2_:N_2_ gas mixture, respectively [11]. The culture medium was removed and replaced daily with the same concentration of antimicrobials for one week. The bacteria were harvested after seven days of incubation and each subsequent passage was performed as previously described [5]. The bacterial growth was monitored using immunoperoxidase staining with specific rabbit polyclonal antibody as described previously [5, 13]. *L. intracellularis* was maintained in the McCoy culture until the number of HICwas 90 to 100%.

After the establishment of a pure culture, each*L. intracellularis* isolate was used to quantify the inoculum for the antimicrobial MIC assay using a previously described staining protocol [5, 13]. Briefly, a series of ten-fold *L. intracellularis* dilutions, from 10^0^ to 10^− 5^, was dilutedin PBS. Then, 10 μl of each dilution was applied into 15-well glass slides as duplicates and the slides were allowed to dry at 37 °C. After fixing with acetone at 4 °C, the slides were stained with the modified immunoperoxidase monolayer assay (IPMA) protocol as described by Guedes et al. [14] using rabbit polyclonal antibody [15]. The lowest dilution that had a*L. intracellularis* quantity between 50 and 500 bacteria/well was counted using a light microscope with 40X objective lens, and the initial concentration was calculated.

### Source and preparation of antimicrobials

For the Brazilian isolates the following antibiotics were used: Chlortetracycline hydrochloride, lincomycin hydrochloride, and tylosin tartrate obtained as pure chemicals from Sigma Aldrich (St. Louis, MO, USA). Tiamulin hydrogen fumarate and valnemulin hydrochloride were supplied as pure chemicals from Novartis Animal Health (Switzerland, Basel). For the Thailand isolates, amoxicillin, Zinc-bacitracin, carbadox, enrofloxacin, gentamicin sulfate, polymyxin B (colistin), spectinomycin dihydrochloride, sulfamethazine and trimetroprim were also used and obtained as pure chemicals from Sigma Aldrich (St. Louis, MO, USA). The combination of lincomycin-spectinomycin was prepared as a 1:1 ratio for determination of the combined activity. The working solutions of tested antimicrobials were prepared as previously described [5]. Briefly, the antimicrobial stock solutions were prepared to a final concentration of 2560 μg/ml and were filtrated through 0.2 μm-pore size filters..A series of two-fold dilutions of the stock solution were made and then further diluted 1:10 with culture medium. The final concentrations of tested antimicrobials were 0.125, 0.25, 0.5, 1, 2, 4, 8, 16, 32, 64, 128 μg/ml. Each concentration of antimicrobial was tested in triplicate and each strain of *L. intracellularis* was tested twice from two independent bacterial passage.

### Antimicrobial sensitivity testing

The MIC assays were performed as described by Wattanaphansak et al. [5]. The antimicrobials used for each strain were chosen according to the use in the pig industry of each country. Briefly, the intracellular and extracellular activities were used to evaluated MICs of antimicrobials against *L. intracellularis*. The intracellular MIC was defined as the effect of antimicrobials on *L. intracellularis *when intracellular organisms were inside of the enterocytes [5]. One hundred μl of bacterial solutionwas added into one-day-old McCoy cells, seeded in 96 well plates. After 24 h of incubation in a sealed bag [16], the bacterial suspension was removed and replaced by 100 μl of fresh culture medium. The antimicrobial suspension was replaced every day for three consecutive days post inoculation.

The extracellular MIC testing was performed as described by Wattanaphansak et al. (5) in order to measure the effect of antimicrobial on *L. intracellularis* when the bacteria were freely in the gut lumen. For this, a series of two-fold dilutions of stock antimicrobial solutions were diluted 1:10 with culture medium which contained *L. intracellularis*. The suspension was incubated at 37 °C in a bag for two hours, allowing the bacteria to be exposed directly to the antimicrobials. After incubation, 100 μl of the bacterial suspension was transferred to one-day-old McCoy cells. The medium was removed 24 h after incubation (under microaerophilic conditions) and replaced with 100 μl of fresh culture medium, without any antimicrobials, for three consecutive days. Each test plate contained control culture, containing no antimicrobials.

After 5 days of incubation for both assays (intracellular and extracellular), supernatant from the infected plates was removed and the cell culture monolayer was fixed with 50 μl of cold 50% acetone and 50% methanol for 1 min. To assess the inhibitory effect of each antimicrobial on *L. intracellularis* proliferation, the infected plates were stained using a modified immunoperoxidase monolayer assay staining method as described previously [5]. Briefly, the fixed plates were re-hydrated with PBS for 30 min. The PBSwas discarded and 50 μl of rabbit polyclonal antibody diluted in skim milk buffer to 1:10,000 was added. After 30 min of incubation at 37 °C, the plates were then washed four times with PBS. Fifty μl of anti-rabbit IgG horseradish peroxidase conjugate diluted 1:5000 in skim milk buffer was added to each well. After incubation for 30 min, the plates were washed four times with PBS. One hundred μl of chromogen solution (500 μl of 3-amino-9-ethyl-carbazol, 9.5 ml of acetate buffer, 5 μl of 30% hydrogen peroxide) were applied and incubated for 20 min. Finally, the stained plates were washed with distilled water and allowed to air-dry.

The infected cells were considered to be HIC if the number of *L. intracellularis* inside the host cells had proliferated to greater than 30 bacteria per cell [1]. A comparison was then made where the number of HICs in each well was expressed as a percentage compared to the average HIC of controls. The intracellular and extracellular MIC endpoints of antimicrobials were defined as the lowest antimicrobial concentration that inhibited 99% of *L. intracellularis* proliferation in the McCoy cells.

## Abbreviations

HIC: Heavily infected cells
IPMA: Immunoperoxidase monolayer assay
MIC: Minimum inhibitory concentration
PBS: Phosphate buffered saline
PE: Proliferative enteropathy
PHE: Proliferative hemorrhagic enteropathy
PIA: Porcine intestinal adenomatosis
